# Sexual signalling in female crested macaques and the evolution of primate fertility signals

**DOI:** 10.1186/1471-2148-12-89

**Published:** 2012-06-18

**Authors:** James P Higham, Michael Heistermann, Carina Saggau, Muhammad Agil, Dyah Perwitasari-Farajallah, Antje Engelhardt

**Affiliations:** 1 Jr Research Group on Sexual Selection, Reproductive Biology Unit, German Primate Centre, Kellnerweg 4, Göttingen, 37077, Germany; 2 Department of Anthropology, New York University, 25 Waverly Place, New York, NY, 10003, USA; 3 Reproductive Biology Unit, German Primate Centre, Kellnerweg 4, Göttingen, 37077, Germany; 4 Faculty of Veterinary Medicine, Bogor Agricultural University, Bogor, Indonesia; 5 Primate Research Center, Bogor Agricultural University, Bogor, Indonesia; 6 Faculty of Mathematics & Natural Sciences, Bogor Agricultural University, Bogor, Indonesia; 7 Courant Research Centre “Evolution of Social Behaviour”, Kellnerweg 6, Göttingen, 37077, Germany

**Keywords:** Fertility signals, Sexual selection, Sexual swellings, Primate

## Abstract

**Background:**

Female signals of fertility have evolved in diverse taxa. Among the most interesting study systems are those of multimale multifemale group-living primates, where females signal fertility to males through multiple signals, and in which there is substantial inter-specific variation in the composition and reliability of such signals. Among the macaques, some species display reliable behavioural and/or anogenital signals while others do not. One cause of this variation may be differences in male competitive regimes: some species show marked sexual dimorphism and reproductive skew, with males fighting for dominance, while others show low dimorphism and skew, with males queuing for dominance. As such, there is variation in the extent to which rank is a reliable proxy for male competitiveness, which may affect the extent to which it is in females’ interest to signal ovulation reliably. However, data on ovulatory signals are absent from species at one end of the macaque continuum, where selection has led to high sexual dimorphism and male reproductive skew. Here we present data from 31 cycles of 19 wild female crested macaques, a highly sexually dimorphic species with strong mating skew. We collected measures of ovarian hormone data from faeces, sexual swelling size from digital images, and male and female behaviour.

**Results:**

We show that both sexual swelling size and female proceptivity are graded-signals, but relatively reliable indicators of ovulation, with swelling size largest and female proceptive behaviours most frequent around ovulation. Sexual swelling size was also larger in conceptive cycles. Male mating behaviour was well timed to female ovulation, suggesting that males had accurate information about this.

**Conclusion:**

Though probabilistic, crested macaque ovulatory signals are relatively reliable. We argue that in species where males fight over dominance, male dominance rank is surrogate for competitiveness. Under these circumstances it is in the interest of females to increase paternity concentration and assurance in dominants beyond levels seen in species where such competition is less marked. As such, we suggest that it may in part be variation in male competitive regimes that leads to the evolution of fertility signalling systems of different reliability.

## Background

The study of inter-sexual communication is central to sexual selection theory [1]. One of the most important aspects of condition that females can signal to males is fertility, and fertility signals have evolved widely throughout the animal kingdom from insects [e.g. paper wasps, [2]] to birds [3], to mammals [e.g. pandas, [4]]. Among the most interesting study systems are those of primates living in multimale multifemale groups, where females signal fertility to males through multiple signals, and in which there is substantial inter-specific variation in the composition and reliability of such signals. Such signals include famous examples such as the anogenital (sexual) swellings of some female primates, the function of which continues to be the topic of much debate [e.g., [5-9]]. The general framework of the socio-ecological model postulates that females distribute themselves in space with respect to food resources, and that males then distribute themselves with respect to the distribution of males [10-12]. These factors therefore create the selective environment in which females evolve to indicate oestrus to males with increasing or decreasing accuracy, and the distribution of females in space and time consequently determines the general likelihood that females will exhibit sexual swellings [8]. One prevailing model for the evolution of these signals is the graded-signal hypothesis, which proposes that swellings represent probabilistic signals of intra-cycle variation in fertility, with swellings being largest when this probability is highest, and smallest when it is lowest, such that ovulation occurs during the maximal swelling period [8]. This model proposes that such probabilistic signals exist in multi-male multi-female groups both to confuse and assure males of paternity. By biasing paternity towards good quality males, females may obtain a variety of indirect and direct benefits, while still enabling sufficient paternity confusion to prevent infanticide from other males who are unlikely (but not impossible) to be fathers.

Over the past decade, several studies of wild and free-ranging primates have tested this evolutionary model, and collectively it has found broad support [e.g. chimpanzees, [13]; Barbary macaques, [14]; olive baboons, [15]; yellow baboons, [16]]. However, some studies have shown apparent inter-specific variability by finding that swellings do not reliably indicate ovulation [e.g. long-tailed macaques, [17]; Assamese macaques, [18]]. At the same time a number of studies have focused on the extent to which female behaviour itself may indicate this timing to males. Both primate sexual swellings [19,20] and female sexual behaviour [reviewed in [21], see also [22-24]] are known to be related to the ovarian hormones oestrogen (positively) and progesterone (negatively), such that both signal types may potentially indicate the timing of ovulation to males. Field studies over the last decade have, as in the case of sexual swellings, found inter-specific variability in relationships between ovulation and female sexual behaviour, with some studies finding that behaviour itself can be considered a probabilistic signal of ovulation [e.g. long-tailed macaques [17]; tonkean macaques, [23]] while other studies did not [e.g. Barbary macaques, [14]; olive baboons [24]].

The fitness benefits to females of employing probabilistic signals of ovulation are that they can offer both paternity confusion and assurance at the same time – allowing high-ranking males who mate with females when conception is most likely a high degree of paternity assurance, while also offering a small probability of paternity to other males who mate when conception is possible but unlikely [8,25]. It has also been suggested that this mechanism might be further elaborated by the use of different signals in different modalities with different transmission differences [24,26]. As such, consorting males may receive extra assurance of their conceptive probability from signals to which other males do not have access [24,26]. Several studies have now shown that male sexual behaviour seems well timed to female ovulation in a probabilistic fashion [chimpanzees, [13,27]; long-tailed macaques, [26]; Barbary macaque, [28]; olive baboons, [24]]. The extent to which males may be influenced by swelling size and/or other cues in combination remains unclear however. In olive baboons, it has been shown that male consort behaviour is well correlated with female sexual swelling size, but that copulatory behaviour within consorts is not, suggesting that other cues may be important to consorting males [24].

One of the most interesting groups in which to study these inter-connected issues are the macaques, some of which show sexual swellings while others do not [8]. Interestingly, the macaques show a great deal of variation in levels of male reproductive skew [e.g. 6-25 % in M. sylvanus, [29-31]; 20-30 % in M. mulatta, [32-34]; 60-90 % in M. fascicularis, [35,36]], and in sexual dimorphism [37]. In species with high dimorphism and reproductive skew, males undergo contest competition for females, fighting for dominance, whereas in species with lower reproductive skew, males undergo scramble competition for females and queue for dominance [38]. The extent to which males compete in contests over dominance may determine the extent to which male rank is surrogate for some aspect of quality or competitive ability as opposed to merely representing group tenure length [34]. As such, high-ranking males may be desirable partners for females in some species, but less so in others. As more reliable signals of ovulation assist dominant males with the monopolization of female fertile periods, it may be in the evolutionary interest of females in species where males compete over dominance to give more reliable signals of ovulation, and females in species where males queue for dominance to give relatively less reliable signals of ovulation. As such, variation in macaque mating systems may be directly related to variation in fertility signalling systems, and greater male reproductive skew and sexual dimorphism should make it increasingly in the female’s interest to offer relatively more reliable signals of ovulation.

Although studies of several macaque species have addressed issues of reproductive skew or the relative reliability of ovulatory signalling, the 7 species of Sulawesi macaque [39], which show the greatest levels of sexual dimorphism [37] (and consistent with this, in crested macaques, accompanying high levels of mating skew, Engelhardt et al. unpublished manuscript), remain poorly studied though see [20] for data on captive Tonkean macaques. Here, we present data on wild Sulawesi crested macaques at Tangkoko, a species of high sexual dimorphism [37], abundant signals of male dominance including bright colours and loud calls [40] and high male mating skew (Engelhardt et al. unpublished manuscript). Our analysis is structured around several explicit aims (Table 1): 1) to assess the extent to which changes in female sexual swelling size and behaviour are related to concentrations of the hormones oestrogen and progesterone; 2) to assess whether changes in female swelling size and behaviour are related to intra-cycle (the timing of ovulation) and inter-cycle (conceptive versus non-conceptive cycles) variation in conceptive probability, and so may potentially indicate this probability to others; 3) to assess whether changes in male behaviour are related to intra- and inter-variation in conceptive probability, and so determine whether males appear to have information about female fertility; 4) to assess the extent to which changes in male behaviour are related to sexual swelling size, and so whether males may respond to this cue in mating decisions. Due to high levels of sexual dimorphism and reproductive skew in this species we predicted that, although signals would be probabilistic in their nature [so still fulfilling paternity confusion roles, 8], female signals of ovulation may nonetheless be more reliable than those seen in related species. Consistent with this, we predicted that males would time their mating behaviour to ovulation relatively accurately.

**Table 1:** Summary of manuscript aims and how they are addressed by different sets of analyses

	**Response Variables**		
Predictor Variables	Swelling Size	Female Behaviors	Male Behaviors
E:P levels	Aim 1	Aim 1	
Day Relative to Ovulation; Cycle Type (conceptive or non-conceptive)	Aim 2	Aim 2	Aim 3
Swelling size			Aim 4

## Methods

### Study site and population

The study was undertaken as part of the Macaca Nigra Project (founded March 2006) at the Tangkoko-Batuangus/Duasudara Nature Reserve at the northernmost tip of Sulawesi (1 o 34’N, 125 o 14’E). The reserve was established in 1980, comprises an area of 8,867 hectares, with a sea boundary of 12 km, and ranges from sea level to an elevation of 1,350 m [41,42]. All research was undertaken between July 2006 and July 2007 on two study groups (Rambo I, Rambo II) which were studied previously by other researchers [43,44]. We re-habituated these groups from April 2006 to July 2006, and identified and named all group members according to individual characteristics such as size, gait, cuts, missing digits, scars etc. The home range of both groups overlapped and included primary forest, secondary forest and, for Rambo II, also gardens near the village. During the study period, both groups ranged in size from 65–70 individuals, with Rambo I consisting of 10 adult males and 21 adult females, and Rambo II 7 adult males and 15 females. In total, data are presented here for 31 conceptive (N = 16) and non-conceptive (N = 15) cycles from 19 females covering 417 observation days and 2,443 hours of behavioural data.

### Faecal samples and hormone analysis

We collected faecal samples for hormonal analysis opportunistically during follows of focal females, with collection occurring on a daily basis at least during mid-cycle, i.e. the period of maximum swelling and the seven following days to allow ovulation to be timed accurately in each cycle [45]. Overall, samples were collected 3–7 times per week in 31 cycles of 19 females. From these samples we were able to determine the timing of ovulation based on patterns of immunoreactive faecal progestogen concentrations measured by enzyme immunoassay (EIA). For this, samples were initially lyophilised and pulverized and an aliquot of the resulting powder was extracted with 80 % methanol in water [46]. Fecal extracts were then analyzed for concentrations of immunoreactive 5α-pregnane-3α-ol-20-one (i5-P-3OH), a group-specific measurement of 5α-reduced 20-oxo pregnanes, which represent abundant progesterone metabolites in faeces of a variety of mammals [for a review, see [47]], including macaques [48]. The assay has previously been applied successfully to monitor female reproductive status and to assess the timing of ovulation in other species of macaques [48,49]. Sensitivity of the assay at 90 % binding was 20 pg/well. Intra- and inter-assay coefficients of variation of high and low value quality controls were 7.8 % and 15.4 % (high) and 9.5 % and 15.7 % (low). The presumed time of ovulation was determined by counting back from the defined post-ovulatory rise in faecal progestogen levels [50]. In this way, a two day ovulation window was determined for all 31 cycles as the 2 and 3 days previous to the post-ovulatory rise in progestogens [as in, e.g., [17]]. To examine the relationship between our measures of female sexual swelling size and female behaviour, fecal extracts were also measured for concentrations of oestrogens using an EIA for the measurement of oestrone (E1C), an abundant oestrogen in macaque feces [51]. The assay was carried out as described elsewhere [52]. Sensitivity of the assay at 90 % binding was 35 pg/well. Intra- and inter-assay coefficients of variation of high and low value quality controls were 5.8 % and 10.3 % (high) and 6.9 % and 13.9 % (low).

### Measuring swellings

To investigate changes in swelling size, female sexual swellings were filmed with a digital video camera (Sony DCR-HC 90E) or were photographed with a digital camera (Canon EOS 350 D) on each observation day. Images were taken from directly behind the focal individual, under good light conditions. Five pictures of each female’s perinea were taken to allow assessment of the size of the ischial callosities, in which we took a second image of a tape measure in the same location as the female with the camera in same position [following e.g., [13,53]]. The camera lens was kept on manual focus for the second image and the tape measure was moved until it was in focus, ensuring a high degree of accuracy in making sure that the tape measure and swelling were photographed at the same distance from the camera. Following this assessment, we used the size of the ischial callosities in all images to assess swelling size, [see [49]]. We measured the height of the left and right callosity in all five images using the measurement methods described below. We then took a mean of these five values.

**Figure 1:**
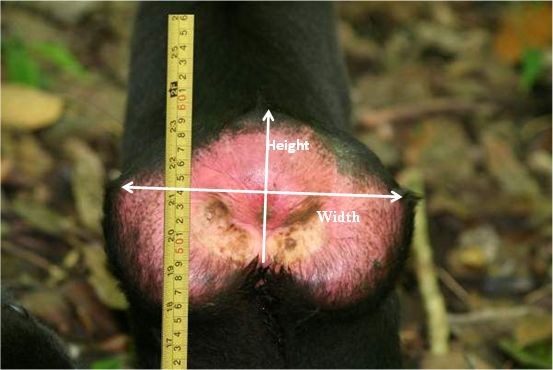
Measurements of swelling size taken in each image.

During each cycle from each female we took an average 24 (range 5–63) swelling images. If the swelling was filmed with the video camera, sequences of the swelling were transferred to a Laptop with Video Capture 6.0 and Scenalyzer 3.52. One picture was captured daily from the video sequences of each observed female with ACDSee 5.0.1. If images were taken using the camera, the highest quality image (if multiple images were available) was chosen and images were transferred to Adobe Photoshop CS2. To correct for differences in camera distance and zoom between images, we adjusted each swelling size measurement for the known size of the ischial callosities (see above). Wherever possible, this was done using the left ischial callosity; if the left ischial callosity was not clearly observable for any reason, the right one was used instead. We measured the ischial callosity height in each image using the Adobe Photoshop measurement tool, and expressed this as a fraction of the known ischial callosity height. We then measured the swelling height, and multiplied this by the ischial collosity fraction to get the true swelling size measurement. We undertook two different measurements of swelling size in this way (Figure 1): swelling width (a horizontal line under the edges of the left and right ischial callosity); and swelling height (a line from the highest point of the swelling down to the line under the ischial callosities).

### Measuring Behaviour

Behavioural data were collected on adult females exhibiting swellings with several Psion Workabout Pro M handhelds (Software: PTab Spreadsheet v.3.0; Z4Soft). Data were collected using instantaneous focal animal sampling [54]. Focal females were followed every day from dawn until dusk, with observation time averaging 5.9 ± 0.1 hours per observation day. We focused our observations and analyses on a number of key socio-sexual behaviours (Table 2).

**Table 2:** Tested behaviours, quantification method and results of analyses

**Sex**	**Behaviour**	**Quantification**	**Pre-Fertile**	**Fertile**	**Post-Fertile**	**P value**	**Relationship to OV**
Female	Approach and solicitation	Count offset for observation time (i.e. rate).	3.11	3.67	1.94	<0.001	+
	Approach and parade (ritualized series of presentations involving passing back and forth in front of the male)	Count offset for observation time (i.e. rate).	0.68	0.79	0.43	<0.001	+
	Lipsmack at male during copulation	Binary (female does or does not).	0.57	0.64	0.51	=0.003	+
	Look at male during copulation	Binary (female does or does not)	0.91	0.91	0.88	p>0.1	None
	Reach back to male during copulation	Binary (female does or does not)	0.38	0.44	0.36	<0.001	+
	Give copulation call during copulation	Binary (female does or does not)	0.22	0.29	0.14	<0.001	+
Male	Ignore female approach	Binary (male does or does not)	0.49	0.47	0.41	*p*=0.009	-
	Inspect female	Binary (male does or does not)	0.45	0.48	0.50	p>0.1	None
	Mount female	Logged rate (n/hr)	2.68	3.56	2.28	P<0.001	+
	Mate with female	Logged rate (n/hr)	2.71	3.52	2.27	P<0.001	+
	Give copulation call during copulation	Binary (male does or does not)	0.41	0.57	0.53	P<0.001	+

Mean values across all cycles are given for the 5 days before the fertile period (Pre-Fertile), the four day fertile period (Fertile) and the five days following the fertile period (Post-fertile). Values were first averaged across all cycles for each day with respect to the ovulation window, then across all days within each respective period. For rate behaviours, values given here are n/hr. For binary variables, values given are proportion of copulations in which the behaviour occurred. P values of the statistical test employed are given (see text for full statistical results).

### Data analysis

As female hormone levels may be related to female behaviour and as, independently of this, female behaviour may indicate the timing of ovulation specifically, we assessed relationships between sexual swelling size and behaviour and both hormone levels, and estimates of the timing of ovulation based on progestogen levels, separately. To do this, we first took the progestogen (P) and estrogen (E) levels measured from our faecal samples and tested whether female sexual behaviour was related to the E:P ratio [e.g. [26]]. When aligning measured E:P to sexual behaviour we had to consider both that there is an excretion delay before hormone levels measured in faeces reflect those measured in blood [e.g. [55]], but also that there may be a delay in response of swelling size and behaviour to changes in blood hormone levels [e.g. [19]]. As such, we aligned hormone levels measured from faeces to measures of swelling size and sexual behaviour all collected on the same day. A previous study that systematically tested different potential alignments found that such an alignment produced significant results between hormone levels and sexual behaviour in female baboons [23].

We tested relationships between the timing of ovulation and swelling size and behaviour using the 14 day period around ovulation only. We created a scale (Day Relative to Ovulation = DayRO) whereby the two days of the ovulation window were both considered Day 0, and each day before (e.g. Day −1, Day −2) and after this (e.g. Day +1, Day +2) were designated accordingly. We did this and analysed data for a period representing the total fertile phase of the cycle [the ovulation window plus the two preceding days to allow for sperm longevity, see e.g. [14]] and a 10 day period surrounding the fertile phase (5 days before, 5 days after). As we do not expect a linear relationship between this scale and measures of swelling size and behaviour, but instead peak around Day 0 with lower values on either side, we squared this scale for analysis [as in [15,24,56,57]]. We classified cycles as conceptive (N = 16) if an infant was born around 6 months later [see [58]], or if it was the last female cycle [either assessed hormonally or by the occurrence of swellings, as *M. nigra* does not exhibit post-conception swellings, see [58]; Engelhardt et al. unpublished manuscript], or if miscarriage was subsequently observed (assessed by female bleeding from the vagina, N = 3). Cycles were classified as non-conceptive (N = 15) if they were immediately followed by another cycle (assessed hormonally or by the occurrence of swellings, as above).

To assess relationships between swelling height and width, and between hormone levels and swelling size (Aim 1), we used general Linear Mixed Models (LMMs) to assess the response of swelling height to variation in a fixed covariate (swelling width or E:P) while controlling for multiple observations of the same females from the same groups (random factors, female ID nested within group). As many of the behavioural variables were not normally distributed but featured a binary response (e.g. either the female gave a copulation call or she did not), Generalized Linear Mixed Models (GLMMs) were used with a binomial error structure and a logit link function. For models of male mating and mounting, rates (n/hr) were normally distributed after log transformation and general LMMs were undertaken. For models of female parading and solicitation rates, values were not normally distributed even after transformation. As such, these variables were treated as counts and modelled using poisson error structures, in which counts were offset for observation time.

In models of female and male behaviour and variation in intra- and inter-cycle fertility (Aims 2 and 3), all models featured a different behavioural variable as the response, DayRO (covariate) and Cycle Type as fixed variables, and female ID nested within group as random factors. For models testing relationships between male behaviour and sexual swelling size (Aim 4), the same model structures were used but with swelling height (covariate) replacing DayRO and Cycle Type as the fixed variable. Statistics were performed in PASW 18.0 (LMMs) and R 2.13.0 (GLMMs). Models of binary response variables were undertaken using the lmer function of the lme4 package [59], while female parading and solicitation rate models were undertaken using the MCMCglmm package [60], as data were overdispersed. All tests were two-tailed and *p* < 0.05 was considered statistically significant.

## Results

**Figure 2:**
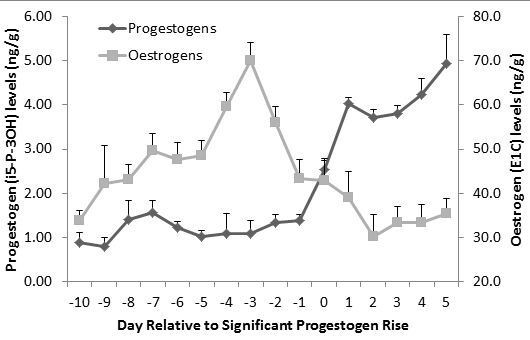
Composite profiles of i5-P-3OH and E1C levels of the 31 cycles included in analyses.

Our hormonal data indicated clear oestrus cycles in the study females, with clear oestrogen peaks occurring 3 days before the post-ovulatory rise in progestogen levels (Figure 2).

**Figure 3:**
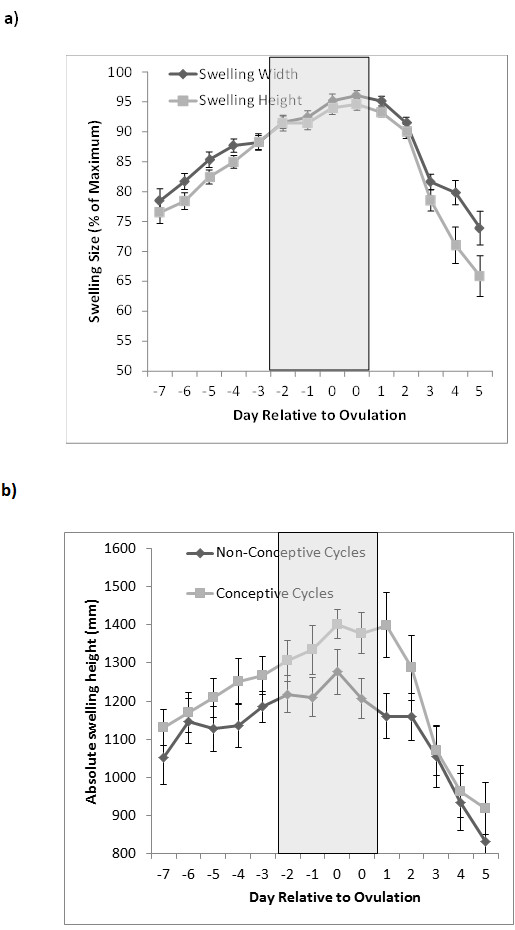
**Swelling size in relation to the timing of ovulation: a) the close agreement between measures of swelling height and width; b) the difference in swelling height between conceptive and non-conceptive cycles.** The grey shaded area represents a presumed four day fertile period that incorporates the two-day ovulation window.

1) Relationship between female hormones and sexual swelling size and female behaviour.Variation in sexual swelling height and width was highly significantly related (F_1,302.0_ = 197.6, *p*<0.001; Figure 3a). For this reason, we used swelling height only in all further analyses. The E:P ratio significantly predicted sexual swelling height, with increasing E:P related to increased swelling size (F_1,271.3_ = 6.546, *p*=0.011). The E:P ratio also predicted some female behaviours, with females more likely to look back (z*=* 4.472, *p*<0.001) and lipsmack (z= 8.806, *p*<0.001) during mating when the E:P ratio was higher. Females also solicited (β = 0.0005192, Lower CI = 0.0001659, Upper CI = 0.0009039, *p*=0.004) and presented (β = 0.0007584, Lower CI = 0.0002467, Upper CI = 0.0012233, *p*=0.002) at higher rates when E:P levels were higher. No other analysed behaviours were significantly related to the E:P ratio.

2) Relationship between intra- and inter-cycle variation in fertility and sexual swelling size and female behaviour.Sexual swelling height was significantly related to DayRO (F_1,289.4_ = 63.685, *p*<0.001, Figure 3a). There was also a significant effect of cycle type, with conception cycles having higher swelling heights (i.e. bigger swellings) for each day with respect to ovulation (F_1,305.9_= 10.877, *p*=0.001; Figure 3b).Most of the analysed behavioural patterns were significantly related to the timing of ovulation (Table 2). Females solicited (β = -0.010326, Lower CI = -0.016677, Upper CI = -0.004106, *p*<0.001), and paraded (β = -0.016278, Lower CI = -0.025348, Upper CI = -0.006279, *p*<0.001) towards males at higher rates closer to ovulation. During mating, females lipsmacked at males (z = -2.966, *p*=0.003), reached back to males (z = -6.125, *p*<0.001) and gave copulation calls (z = -7.205, *p*<0.001) significantly more often closer to ovulation.Three female behavioural patterns showed a statistically significant difference between conception and non-conception cycles. Females lipsmacked (z = -3.655, *p*<0.001), gave copulation calls (z = -2.662, *p*=0.008) and looked back significantly more often in non-conception cycles than in conception cycles (z = -2.225, *p=*0.026). However, mean (across all cycles) differences in these three behavioural variables between the two cycle types were less than 10%.

3) Relationship between male behaviour and intra- and inter-cycle variation in fertility.Several analysed male behavioural patterns showed a significant correlation to the timing of ovulation (Table 2). Crucially, males mounted (*t*_*1,321*_= -5.077, *p*<0.001) and mated (*t*_*1,322*_= -5.050, *p*<0.001) with females more often closer to ovulation. Males also ignored female approaches significantly less often closer to ovulation (z = 2.587, *p*=0.009) and were more likely to give a copulation call during mating (z = -9.106, *p*<0.001) when ovulation was approaching. Two male behavioural patterns showed a significant difference between conception and non-conception cycles. Males ignored female solicitations significantly less often in conceptive cycles (z = -2.814, *p*=0.004), and gave copulation calls significantly less often in conceptive cycles (z = -7.915, *p*<0.001). However, mean (across all cycles) differences in these three behavioural variables between the two cycle types were less than 10%.

4) Relationship between male behaviour and sexual swelling size.The key male behaviours of mounting and mating rates were both significantly positively related to sexual swelling height (mounting, t_1,265_=3.552, *p*<0.001; mating, t_1,265_=3.577 *p*<0.001). Interestingly, both of these effects were independent of variation in intra-cycle fertility. As such, in models containing both DayRO and swelling height, both variables emerge as independently significant factors influencing male mounting (swelling height, t_2,264_=2.531, p=0.012; DayRO, t_2,264_= -2.098, *p*=0.037) and mating (swelling height, t_2,264_=2.537, *p*= 0.012; DayRO, t_2,264_= -2.171, *p*=0.031) rates. No other behaviours were significant related to swelling height (all *p*>0.1).

## Discussion

Our results show that crested macaque females give signals of ovulation that are probabilistic in nature, but that are relatively clear compared to those shown by other multi-male multi-female group-living catarrhines. Unlike in many previous studies of other primate species, where sexual swellings but not behaviour indicated the timing of ovulation [e.g. Barbary macaques, 14; olive baboons, [15,24]], or where behaviour but not sexual swellings indicated ovulation [e.g. long-tailed macaques, [17]], measures of both sexual swelling size and sexual behaviour indicated intra-cycle variation in fertility, potentially indicating the timing of ovulation to males. The only other species yet studied which may show this is the very closely related Tonkean macaque [23], although fine-scale analyses have not yet been undertaken. Significant behavioural measures include the frequency of copulation calls. To our knowledge this is the first time that measures of copulation calls have been shown to vary specifically with respect to ovulation, adding a further modality to the range of signals to which crested macaques may indicate ovulatory timing. Consistent with the idea that males received reliable information about female reproductive status, numerous key male sexual behaviours were well timed to the most fertile part of the cycle. These data add to those of several other catarrhine species that suggest that males may be able to time mating effort to female ovulation [e.g. long-tailed macaques, [26]; chimpanzees, [13,27]; Barbary macaques, [28]; olive baboons, [24]]. Despite this however, crested macaque females are nonetheless still probabilistic signallers, with swellings expressed over multiple days that extend beyond the fertile phase. As such, their swellings are still consistent with the graded-signal framework [8].

Our analyses of variation in inter-cycle fertility revealed significant differences between conceptive and non-conceptive cycles. Females expressed significantly larger sexual swellings in conceptive cycles than in non-conceptive cycles, and also exhibited some behaviours less often in conception cycles (though behavioural differences were for only a few variables, and were less than 10 % different between cycle types). Males showed some behavioural differences between cycle types, but consistent with results in some other species [e.g. chimpanzees [13]], males did not show differences in mating rates between conceptive and non-conceptive cycles. Other analyses from our dataset focusing on mating skew show that alpha males specifically may respond to conceptive cycles to a greater extent than non-conceptive cycles in more functionally important behaviours (Engelhardt et al. unpublished manuscript). Given the strong mating skew and limited reproductive opportunities for lower ranked males seen in this species (Engelhardt et al. unpublished manuscript), males may not mate at higher rates in conceptive than non-conceptive cycles because for most males, all opportunities for mating must be taken. Another possibility is that alpha males, who dominate consortships during fertile phases (Engelhardt et al. unpublished manuscript), have increased information about female cycle status not available to all males. Similarly, only group resident male chacma baboons consorted more with females during conceptive cycles [61] consistent with a potential role for close access [24,26] and familiarity [62] for males in interpreting female primate fertility signals.

The apparent relative reliability of female ovulatory signalling is consistent with the low female oestrus synchrony, high mating skew (Engelhardt et al. unpublished manuscript) and high sexual dimorphism [47] found in this species, as well as the large canines, bright colours and loud calls that have evolved in males [40]. Such evidence indicates both that males are able to exert a high degree of control over female reproduction, and that males engage in contest competition for females, and fight for dominance. Under such circumstances, male dominance rank may be reliably related to male competitive ability [unlike in say, rhesus macaques, where it may be primarily related to group tenure length; [63]], such that high ranking males may be the preferred partners of females. As clearer signals of ovulation further enable male monopolization of female fertile periods by dominant males, it may only be in the evolutionary interest of females to give clearer signals under such circumstances. Females are more likely to derive indirect benefits such as “good genes” [64] from high ranking males in these types of competitive male-male environments. Intra-group direct benefits such as food tolerance [64] should be available from high ranking males in all regimes, but other direct benefits such as predator protection should be more available from high-ranking males where rank is achieved through male strength rather than through queuing.

Our analysis of relationships between male behaviour and sexual swelling size indicate that male behaviour was well timed to this reliable signal of the timing of ovulation. Interestingly male behaviours such as inspection were not related to the timing of ovulation [similar to long-tailed macaques, [17]]. The prevailing evolutionary model for sexual swelling function is one in which females offer unreliable and only probabilistic signals of ovulation which allows females to offer some paternity probability to numerous males, while nonetheless offering the most assurance to the dominant male [8]. It has also been suggested that females may further elaborate this mechanism by using multi-modal signals with different active spaces, in which females can simultaneously offer different males different information about ovarian function [24,26]. Under such a theoretical system, females may offer less reliable signals such as sexual swellings to all males, while making more reliable signals of ovulation such as olfactory signals only available to males with close access to the female [24,26]. Interestingly, this concept seems not to hold for our study species. As we have argued above, the reason for this may be that in species with low oestrus synchrony, high mating skew, monopolized mating and contest competition for dominance, there may be increased advantages to females of signalling ovulation relatively more reliably, and of placing an increased emphasis on biasing paternity towards the dominant. As such, our data provide further evidence that the functional role of sexual swellings is unlikely to prove ubiquitous across all species.

## Conclusion

Our data provide new insights into the evolution of female fertility signals in a taxa in which there is a high degree of variation in the reliability of such signals. Unlike in many multimale multifemale group-living primate species where signals of ovulation appear relatively unreliable, females in this very sexually dimorphic macaque species signal differences in intra- and inter-cycle conceptive probabilities using different types of signal, in this case sexual swellings and female behaviour. Male crested macaques appear to have information about the timing of female ovulation, and time their mating effort appropriately. Together with data on mating skew and the monopolisation of females by dominant males (Engelhardt et al. unpublished manuscript), the present data help build a picture of a highly interesting species in which dominant males fight for rank, monopolize mating and reproduction, and in which females signal fertility to males in a probabilistic but relatively (compared to related species) reliable fashion. Collectively, our data are important for understanding the evolution of female fertility signals because they represent an extreme end of the variation seen across the macaques, some of which do not possess clear signals of ovulation such as sexual swellings at all. Our suggestion that comparative variation in male competitive regimes may be linked to the evolution of fertility signalling systems of different reliability provides testable predictions for other species. Further understanding the selective pressures that have given rise to such comparative variation will provide a wealth of insight into the veracity of current socio-ecological models, as well as our understanding of signal evolution.

## Competing interests

The authors declare that they have no competing interests.

## Author contributions

JH processed data, undertook analyses, drew the figures and wrote the paper; MH provided reagents, oversaw the hormonal analyses and helped draft the manuscript; CS processed data and undertook analyses; MA co-conceived the project, oversaw processing of hormone samples and provided logistical and institutional support in Indonesia; DP-F co-conceived the project and provided logistical and institutional support in Indonesia; AE conceived the project, collected and processed data and co-wrote the paper. All authors read and approved the final manuscript.
